# Carbohydrate Moieties and Cytoenzymatic Characterization of Hemocytes in Whiteleg Shrimp* Litopenaeus vannamei*

**DOI:** 10.1155/2016/9032181

**Published:** 2016-10-19

**Authors:** Norma Estrada, Edwin Velázquez, Carmen Rodríguez-Jaramillo, Felipe Ascencio

**Affiliations:** ^1^Programa Cátedras CONACyT, Centro de Investigaciones Biológicas del Noroeste, S.C. (CIBNOR), 23090 La Paz, BCS, Mexico; ^2^Laboratorio de Patogénesis Microbiana, Centro de Investigaciones Biológicas del Noroeste, S.C., 23090 La Paz, BCS, Mexico; ^3^Laboratorio de Histología e Histoquímica, Centro de Investigaciones Biológicas del Noroeste, S.C., 23090 La Paz, BCS, Mexico

## Abstract

Hemocytes represent one of the most important defense mechanisms against foreign material in Crustacea and are also involved in a variety of other physiological responses. Fluorescent lectin-binding assays and cytochemical reactions were used to identify specificity and distribution of carbohydrate moieties and presence of several hydrolytic enzymes, in hemocytes of whiteleg shrimp* Litopenaeus vannamei*. Two general classes of circulating hemocytes (granular and agranular) exist in* L. vannamei*, which express carbohydrates residues for FITC-conjugated lectins WGA, LEA, and PNA; UEA and Con-A were not observed. Enzymatic studies indicated that acid phosphatase, nonspecific esterase, and specific esterases were present; alkaline phosphatase was not observed. The enzymes and carbohydrates are useful tools in hemocyte classification and cellular defense mechanism studies.

## 1. Introduction

In crustacean decapods, the defense system relies on humoral and cellular mechanisms, with cellular defense coordinated by circulating hemocytes [1]. Hemocytes in crustaceans also are known to be involved in rapid sealing of wounds to prevent loss of hemolymph and prevent infection [2, 3]. Many studies of morphology, structure, function, and classification of hemocytes in decapods indicate three fundamental types, according to the number and size of granules present: hyaline cells, small-granule cells, and large-granule cells [4]. These types are described for penaeid shrimp [5, 6] and freshwater crayfish [7, 8]. Hemocytes can recognize and eliminate or sequester invading pathogens through phagocytosis, encapsulation, and secretion of lysosomal enzymes and bacteriostatic substances [9, 10]. Principally, granular hemocytes carry out phagocytosis by engulfing small foreign particles. Granules are known to contain many enzymes, such as lysozymes, esterases, phosphatases, phospholipases, peroxidases, and proteases and also oxidative enzymes that help to eliminate the foreign material [11–14]. Hemocytes play an important role in producing and discharging agglutinins, such as lectins [15] and antibacterial peptides [16]. Invertebrate hemocytes also have several carbohydrate moieties that act as receptors for invading pathogens, where binding of lectins and carbohydrates leads to structural changes of the complex that induce activation of hemocytes [17, 18].

The Pacific whiteleg shrimp,* Litopenaeus vannamei* (Boone, 1931), is one of the most important farmed species in the world. Economic losses due to disease in shrimp aquaculture have made it necessary to increase our knowledge of the invertebrate immune system. Enzyme cytochemistry has often been used in functional characterization of hemocytes [9]. Differentiating hemocytes with lectins to study specificity and distribution of carbohydrate moieties has been useful in classifying hemocytes and defining their function [18]. To study hemocytes in* L. vannamei* in a basal state for a comprehensive description for the structure and function of the hemocytes, we used microscopic analysis, enzymatic activity by cytochemistry, and glycoconjugates with lectin probes in cells.

## 2. Material and Methods

### 2.1. Extraction of Hemolymph

Juvenile shrimp (average length = 10.3 ± 2.0 cm). Before experiments, the shrimp were kept in laboratory under controlled conditions in a recirculating system in 1500 L fiberglass tanks with 1 *μ*m filtered seawater at 35 PSU and 25°C at pH 8, with constant aeration, and fed commercial pellet feed daily. Hemolymph was extracted with a 27-gauge syringe at the junction between the basis and ischium of the fifth walking leg. Prior to bleeding, the sample area was wiped with 70% ethanol. The syringe contained an equal volume of citrate/EDTA anticoagulant composed of 0.45 M NaCl, 0.1 M glucose, 30 mM sodium citrate, 26 mM citric acid, and 10 mM EDTA at pH 5.4 and put on ice [19].

### 2.2. Type of Hemocytes and Total and Differential Count

Total hemocytes counts were measured with an electronic particle counter (Multisizer, Beckman Coulter, Brea, CA). Cellular viability of each sample was estimated in Neubauer chambers with a fluorescence microscope, after adding a solution of propidium iodide, which is not membrane permeable and usually excluded from viable cells. It is commonly used to label dead cells and as a counterstain in multicolor fluorescent techniques. Hemocytes were more than 90% viable. To determine the type of hemocytes, we counted types of hemocytes in histological sections. For this procedure, we pooled hemolymph mixed at 1 : 1 with Karnovsky's fixative [20] for 24 h at 4°C, washed in several changes of Karnovsky's buffer, dehydrated through an ethanol series, and embedded in catalyzed acrylic monomer (JB-4 plus embedding kit, Polysciences, Warrington, PA). Histological samples were sectioned to 1 *μ*m and stained with hematoxylin-eosin and May-Grünwald Giemsa. Permanent slides were examined under a microscope, and digital photographs were taken. Digital images were used to analyze at least 500 hemocytes at 40x (10 pictures in 10 random fields per slide) (Image-Pro Plus v4, Media Cybernetics, Bethesda, MD, USA), identifying small-granule cells (SGC), large-granule cells (LGC), and hyaline cells (HC), according to Heng and Lei [21]. The percentage of each type of cell was calculated, based on the total number of cells. Data were tested with ANOVA with the* post hoc* Tukey test to test for differences among hemocyte types and staining methods. Numerical data are represented as mean ± SD, using the SPSS 16.0 software (IBM SPSS, Armonk, NY). Results were considered significant at *P* < 0.05.

### 2.3. Lectin-Binding Assays

Differences in the distribution of hemocyte oligosaccharides were studied using five fluorescein isothiocyanate- (FITC-) labeled lectins. The lectins are listed in Table 1. Aliquots of 100 *μ*L of pooled hemolymph (1 × 10^6^ cells mL^−1^) were placed in 0.7 *μ*L polypropylene tubes in triplicate and 5 *μ*L formaldehyde was added to each tube for 30 min. Solutions of the lectins were prepared in their respective buffer to concentrations of 100, 50, 25, and 10 mg lectin mL^−1^. To each tube 100 *μ*L of a lectin solution was added and incubated in the dark for 1.5 h at room temperature. The same procedure was performed for all lectins. After incubation with labeled lectins, the hemocytes were washed and resuspended in 100 *μ*L filtered PBS at pH 7.2 for analysis in a Neubauer chamber with an epifluorescence microscope, using the appropriate filters (Olympus BX41, Tokyo, Japan) at 40x. For the controls, each lectin was incubated in its appropriate competing sugar at a 0.2 M final concentration (see Table 1) for 30 min at room temperature. This solution was then centrifuged for 15 min at 16,000 ×g and hemocytes were incubated in the supernatant for 1.5 h at room temperature. Then, tissues were rinsed in PBS containing the competing sugar and prepared for fluorescence microscopy, as described below. Other control slides were incubated with PBS without lectins. For each treatment, digital images were analyzed (at least 500 hemocytes, 10 pictures in 10 random small Neubauer chamber squares per sample), counting fluorescent and nonfluorescent hemocytes. Intensity and area covered by fluorescence were determined with an image analyzer (Image-Pro Plus v4, Media Cybernetics, Bethesda, MD) at 40x. The percentage of positive (fluorescing) cells per sample was calculated, as defined in reference to total hemocyte number. Multiple independent samples were analyzed to ensure reproducibility. Data were tested with ANOVA with the* post hoc* Tukey test to test differences among different lectins. Numerical data are represented as mean ± SD, using the SPSS 16.0 software. Results were considered significant at *P* < 0.05.

### 2.4. Enzyme Cytochemistry

Hemocytes were prepared by cell adhesion for histochemistry of enzymes. After withdrawing hemolymph, 150 *μ*L was placed on glass slides at densities of 5 × 10^6^ cells mL^−1^ and allowed to adhere for 1 h at 25°C under sterilized moist chamber conditions. The slides were then carefully washed with PBS at pH 7.2 and stained with reagents for a range of lysosomal enzymes. Adhered cells were fixed according to each enzyme tested. Diagnostic kits were used to test for four hydrolytic enzymes, carried out according to the manufacturer's instructions: (1) acid phosphatase (naphthol AS-BI phosphoric acid substrate, #386-A, Sigma, St. Louis, MO), (2) alkaline phosphatase (naphthol AS-BI alkaline substrate, #86-C, Sigma), (3) nonspecific esterase (*α*-naphthyl acetate substrate, #91-A, Sigma), and (4) specific esterase (naphthol AS-D chloroacetate, #91-C, Sigma). For acid phosphatase, duplicate films are treated with L(+)-tartrate-containing substrate; cells containing tartaric acid-sensitive acid phosphatase are devoid of activity. Slides were rinsed in deionized water, allowed to air-dry, and studied microscopically, using an oil immersion lens. For each treatment, digital images were used to analyze at least 500 hemocytes (10 pictures in 10 random fields per slide), identifying positive and nonpositive cells. For all enzymes, the percentage of positive cells was calculated, based on the total number of cells. Control slides were incubated only in buffer; all solutions were made immediately before use. Following this, observations were made with a light microscope. Multiple independent samples were analyzed to ensure reproducibility (*n* = 9 slides from three independent, pooled samples for each technique). Data were tested with ANOVA with the* post hoc* Tukey test to test differences among different enzymes. Numerical data are represented as mean ± SD, using the SPSS 16.0 software. Results were considered significant at *P* < 0.05.

## 3. Results

**Figure 1:**
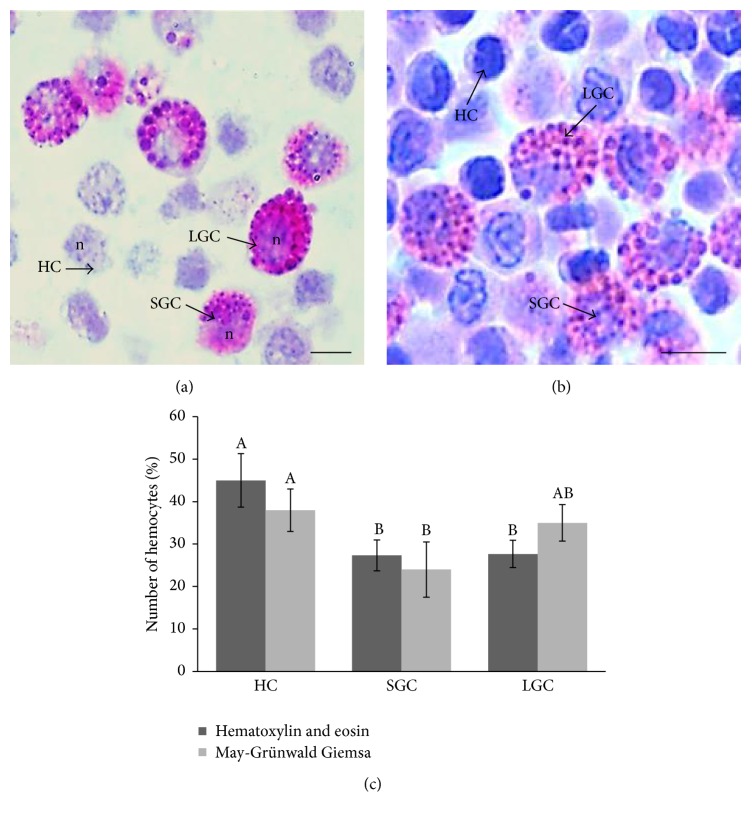
Hemocytes of* Litopenaeus vannamei*. (a) Micrographs of hemocytes stained with hematoxylin-eosin in resin histological sections and (b) hemocytes stained with May-Grünwald Giemsa, showing three types of hemocytes: small-granule cells (SGC), large-granule cells (LGC), and hyaline cells (HC). Scale bar = 5 *μ*m. (c) Differential hemocyte count (DHC) in histological resin sections stained with hematoxylin-eosin and May-Grünwald Giemsa. The relative percentage of DHC was calculated by observing digital images analyzing at least 500 hemocytes. Data represent mean ± SD. Letters indicate significant differences (*P* < 0.05) between hemocyte types. n = nucleus.

Hemocytes stained in histological sections, with hematoxylin-eosin and May-Grünwald Giemsa dyes, showed many cytoplasmic granules of different sizes, as well as cells without granules (Figures 1(a) and 1(b)). Description of hemocyte types using morphological criteria previously developed for the shrimp [21] shows three basic cell types: small-granule cells (SGC), large-granule cells (LGC), and hyaline cells (HC).  Figure 1(c) shows the percentage of different hemocyte types per sample, calculated with reference to the total number of hemocytes.

**Figure 2:**
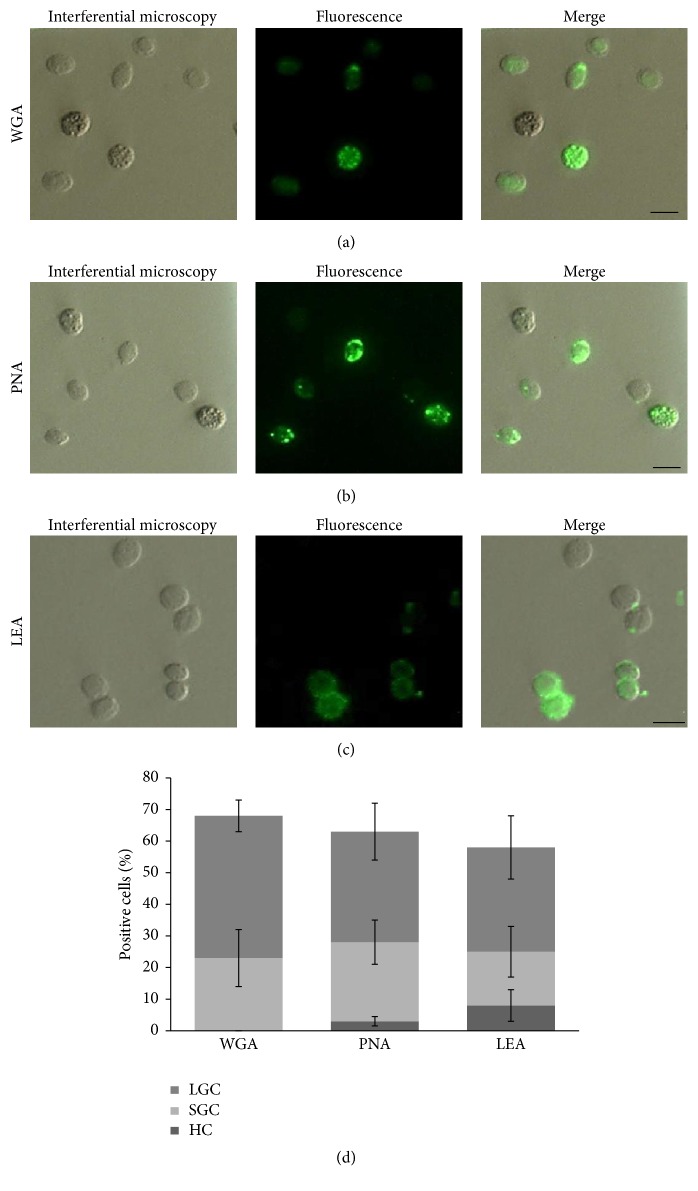
Labeling pattern of fixed hemocytes isolated from* Litopenaeus vannamei* after incubation with FITC-conjugated lectins. Many hemocytes express ligands differentially: (a) wheat germ agglutinin (WGA), (b) peanut agglutinin (PNA), and (c)* Lycopersicon esculentum* agglutinin (LEA). Hemocytes were evaluated by interference contrast microscopy and epifluorescence microscopy. Scale bar = 5 *μ*m. (d) Percentage of total hemocytes and cell types of positive cells labeled by FITC-conjugated lectins. For each treatment, digital images were used to analyze at least 500 hemocytes. The percentage of positive (i.e., fluorescing) cells per sample was calculated, as defined with reference to the total hemocyte number. Data represent mean ± SD.

Binding of five lectins with different carbohydrate specificities to hemocytes of* L. vannamei* was studied using FITC-labeled lectins (Table 1). Circulating hemocytes isolated from* L. vannamei* were able to bind to WGA, PNA, and LEA (Figures 2(a), 2(b), and 2(c)); carbohydrate moieties for UEA and Con-A were not observed.  Table 2 shows whether fluorescence was present in clump of cells or individual cells and shows optimal concentration of lectin for labeling assays. The percentage of positive cells per sample was calculated, defined with reference to the total number of hemocytes, along with percentages of hyaline, small-granule, and large-granule hemocytes (Figure 2(d)). There were no statistical differences in the expression of carbohydrates on the hemocytes. Figures 2(a) and 2(c) and Table 2 show that WGA and LEA reacted with hemocytes of* L. vannamei* in agglutinated and individual cells, with clearly dotted staining of different sizes with WGA, and with LEA also dotted structures were observed, along with uniform label pattern in many cells which seems to label surfaces. PNA was present in high percentages in* L. vannamei* hemocytes also in dotted structures (Figure 2(b)). Known inhibiting carbohydrate residues were used to inhibit labeling of lectin-treated hemocytes. Controls with the inhibitory saccharides demonstrated that the binding of FITC-lectin was specific, since the presence in the incubation medium of the appropriate haptenic sugar (Table 1) abolished or markedly decreased fluorescence.

**Figure 3:**
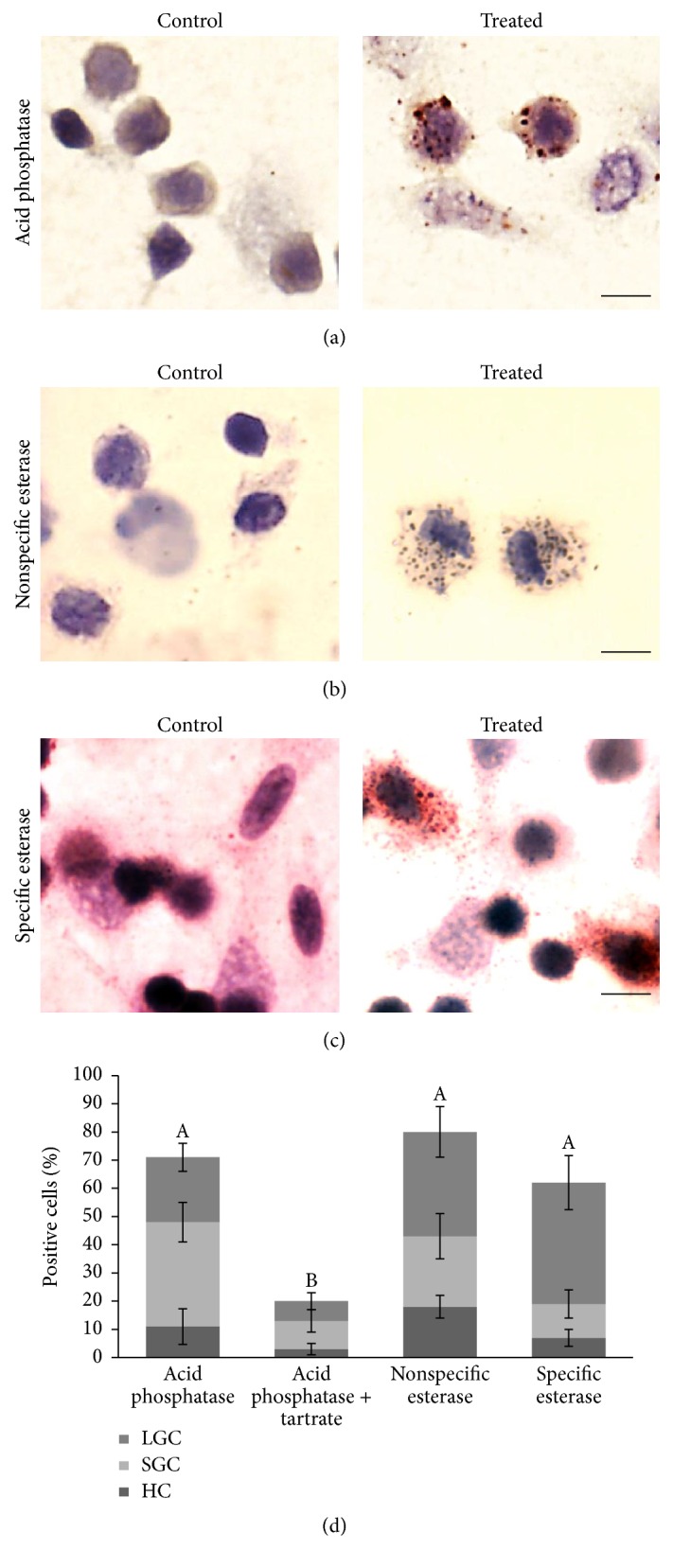
Enzyme cytochemistry of adhered hemocytes isolated from* Litopenaeus vannamei*. The first column of micrographs corresponds to the control cells incubated without the enzyme substrate and the second column shows enzymatic activities. (a) Acid phosphatase, (b) nonspecific esterase (alpha-naphthyl esterase), and (c) specific esterase (AS-D chloroacetate esterase). Scale bar = 5 *μ*m. (d) Percentage of adhered hemocytes of* L. vannamei*, which react with the substrates for different enzymes, along with cell types of positive cells. Data represent mean ± SD. Letters indicate significant differences (*P* < 0.05) in percentage of positive cells.

Hemocytes from* L. vannamei* were analyzed by cell adhesion for several hydrolytic enzymes. Of the four enzymes that we studied, only three were present: acid phosphatase, *α*-naphthyl acetate (nonspecific esterase), and naphthol AS-D chloroacetate (specific esterase) (Figures 3(a), 3(b), and 3(c)). Distribution and location of these enzymes were not homogeneous in the hemocytes; however, strong reactions were observed when positive. Acid phosphatase is clearly localized as patchy structures of different sizes, and nonspecific and specific esterase activities are also found in dotted structures, similar to those found with acid phosphatase activity.  Figure 3(d) shows shrimp hemocytes with positive enzyme activities, along with percentages of hyaline, small-granule, and large-granule hemocytes. A high percentage of hemocytes were positive for acid phosphatase; many hemocytes were resistant to tartrate. A high percentage of* L. vannamei* hemocytes were positive for nonspecific esterase, and also a significant percentage of hemocytes were positive for specific esterases. Reaction sites demonstrating hydrolytic enzymes were less abundant in hyaline cells and more abundant in granulocytes.

## 4. Discussion

There are many studies of morphology, structure, function, and classification of hemocytes in arthropod decapods that show two fundamental types of hemocytes in hemolymph: granular and hyaline types [5, 9, 21, 22]. Hose et al. [23] and Kondo et al. [24] show that hemocytes of the Penaeidae shrimp comprise hyaline cells (HC), small-granule cells (SGC), and large-granule cells (LGC). In our results with hematoxylin-eosin, 45% were HC, 27% were SGC, and 28% were LGC, whereas with May-Grünwald Giemsa 38% were HC, 24% were SGC, and 35% were LGC. These results are different compared to studies of* L. vannamei* and other shrimp species, where the most abundant cells are the SGC, ~40–60% [5, 21, 25]. Small-granule cells are the only hemocyte type involved in all the four known biological functions (phagocytosis, encapsulation, cytotoxicity, and prophenoloxidase activity), while hyaline and large-granule cells are only involved in some of these functions [1]. It is common to use other classification schemes, such as size and shape of the cells. These schemes have inadequacies. Separation of hemocytes in this study using resin histological sections is an adequate method because we can count more than 500 hemocytes and clearly identify them with classical hematoxylin-eosin or May-Grünwald Giemsa staining, an easy way to avoid tedious techniques due to fragility of the cells. Different counts were recorded by relative percentage of the hemocytes in different decapod species having many variations. Hose et al. [26] found that* Sicyonia ingentis* had 50–60% HC, 30% SGC, and 10% LGC. Kakoolaki et al. [27] found that* F. indicus* had 10–15% HC, 20–25% LGC, and 60–65% SGC. In addition, in our study total hemocyte count (THC) is about 6–10 × 10^6^ cells mL^−1^, similar to other works of Penaeidae shrimp [5, 27, 28]. However variation of THC also has been usually reported by researchers. Total and differential hemocyte counts can vary greatly in response to infection, environmental stress, and endocrine activity during moulting cycle [29–31].

We characterized hemocytes of* L. vannamei* with cytochemical tests. The combination of many cytochemical tests is suggested for classification of shrimp hemocytes [9]. Differentiating hemocytes using lectins to recognize glycoconjugates and the activity of diverse hydrolytic enzymes can be useful for classification of different cell types. The method provides information on the function and relationships between cell types. Hemocytes of crustaceans have binding sites for a number of lectins as constituents of glycoproteins and glycolipids in the cell [18, 32]. Our results confirm that circulating hemocytes express ligands (carbohydrates) for lectin-binding in a very heterogeneous manner. The percentage of lectin-labeled hemocytes in* L. vannamei* is similar to WGA and LEA. The WGA lectin selectively binds to N-acetyl-glucosamine (GlcNAc) and to N-acetylneuraminic acid (sialic acid) residues of glycoproteins and glycolipids. The labeling pattern with WGA appears as dotted cytoplasmic structures which correspond to granules present in cytoplasm of many hemocytes of decapods and these are granular hemocytes [18, 32] and are distinct from hyaline hemocytes with fewer or without granules. LEA showed a homogeneous distribution on the cell surface; although this tomato lectin is specific for oligomers of *β*-(1,4)-linked N-acetyl-D-glucosamine, with the binding site being able to accommodate up to 4 carbohydrate units, also LEA binds well to sialoglycoproteins of the membrane. Comparisons have been made between several lectins that share a similar specificity, including WGA, showing that their reactivity with glycoproteins varies. The tomato lectin does not seem to require that the GlcNAc residues be consecutive, a finding that has not been noted for WGA.

Lectin-labeled hemocytes in* L. vannamei* with PNA bound the carbohydrate sequence galactosamine-*β*(1-3)-N-acetyl-D-glucosamine and showed a similar dotted pattern as WGA, but PNA and WGA recognize different carbohydrate determinants; thus the similarity in fluorescence patterns indicates that carbohydrate moieties that are recognized by these two lectins are shared by the same glycoproteins or occur as different glycoproteins that are closely associated. This suggests that the granule hemocytes in* L. vannamei* possess a cocktail of glycoconjugates, whereas LEA recognizes surface membrane receptors of hemocytes. Lectins are widely used to study recognition of carbohydrates by proteins in model systems to understand the molecular basis of how proteins recognize specific terminal sugars or groups of sugars in glycoproteins and glycolipids because they are relatively easy to obtain and have a wide variety of sugar specificities. Lectins assume a particular significance because they act as pattern-recognition proteins and recognize specific carbohydrate moieties on surfaces of pathogen cells that cause agglutination and facilitate binding of foreign particles to and promote ingestion by phagocytes [15, 33, 34]. Lectin-carbohydrate binding leads to a structural change of the complex that induces hemocyte activation [17].

To identify and characterize subpopulations of hemocytes, we used enzyme cytochemistry. Specific esterase and nonspecific esterase were detected in hemocytes from* L. vannamei*, as in similar organisms [9].* L. vannamei* hemocytes were not positive for alkaline phosphatase. The lack of alkaline phosphatase is reported in other crustaceans [9] but had been detected in mud crab,* Scylla serrata* [35]. Since we tested hemocytes in the basal state, production of this enzyme could be induced by external agents. However, hemocytes of* L. vannamei* had a high percentage of cells that were positive for acid phosphatase. The presence of lysosomes is commonly corroborated with acid phosphatase staining in hemocytes and serum of crustaceans [9]. Many cells were tartrate resistant, which indicates that there are cells that have a similar enzyme to human 5-acid phosphatase (Acp5) that is present intracellularly in many cell types, is secreted* in vitro* by macrophages, and participates in phagocytosis of bacteria [36, 37]. In* L. vannamei*, the percentage of cells that are positive for esterases and acid phosphatase was almost the same, 60–80% of the total hemocyte population. This probably reflects the number of granular cells obtained using other methods of detection, such as WGA-binding to granules in hemocytes. The dominance of granular hemocytes in many crustaceans suggests that they are capable of active defense reactions and might be phagocytic cells that contain abundant hydrolytic enzymes and other proteins [4, 23, 38, 39]. Hyalinocytes show limited phagocytic ability and lower levels of hydrolytic enzymes, probably having other functions that are different than phagocytosis, such as use of nutrients or coagulation [27, 40].

In summary, it is interesting to note that most invertebrates possess subpopulations of granular and hyaline hemocytes that can be distinguished with classical staining and microscopic methods. Although recent research has expanded the physiological roles played by decapod hemocytes, extension of this information from one species to another is difficult because there is no unified classification of hemocytes of either group, principally because different techniques are used and the objectives of investigation are different. The immune function of* L. vannamei* is partly based on a sugar code, matching glycan diversity with the presence of lectins and various glycan epitopes identified as ligands to destroy foreign organisms. Lectin-binding staining properties and the presence of lysosomal enzymes seem to be good markers to provide valuable information on hemocyte morphofunctional characteristics. Understanding the immune systems of crustacean decapods is necessary to assess the relative contribution of environmental, anthropogenic, and pathological stresses. Morphological and functional characterization of hemocytes in* L. vannamei* provide some insights into the response of the immune system, yet more work is necessary to identify markers that will facilitate our understanding of hemocyte characteristics.

## Figures and Tables

**Table 1:** Lectins used to investigate cell surface oligosaccharides domains in hemocytes and their competitive sugars used as controls for lectin-binding.

Lectin	Latin name	Source	Resuspension solution	Specificity sugar	Inhibition^*∗*^
Con-A	*Canavalia ensiformis* (Sigma, C7642)	Jack bean	NaCl 0.9% pH 6.5 with 5 mM CaCl_2_	a-D-Mannose, a-D-glucose	Glucose
WGA	*Triticum vulgaris* (Sigma, L4895)	Wheat germ	PBS pH 7.4	(D-GlcNAc), NeuNAc	N-Acetylglucosamine
PNA	*Arachis hypogaea* (Sigma, L7381)	Peanut	PBS pH 7.4	*β*-Galactose(1-3) galNAc	Galactosamine
UEA-I	*Ulex europaeus* (Sigma, L9006)	Gorse, furze	NaCl 0.9%	a-L-Fucose	Fucose
LEA	*Lycopersicon esculentum* (Sigma, L0401)	Tomato	*∗∗*	(glcNAc)_3_	N-Acetylglucosamine

GalNAc = N-acetyl-galactosamine, GlcNAc = N-acetyl-glucosamine, and NeuNAc = N-acetylneuraminic acid.

^*∗*^Competing sugar used in the study at 0.2 M.

^*∗∗*^10 mM HEPES, 0.15 M NaCl, 0.1 mM Ca^2+^, 0.08% sodium azide, and 5 mg mL^−1^ *β*-cyclodextrin.

**Table 2:** Fluorescent lectin-binding.

Lectin	*Litopenaeus vannamei*	
	Lectin-binding	Optimal concentration^*∗*^ (*μ*g mL^−1^)
Con-A	—	100
WGA	C, S	25
PNA	S	50
UEA-I	—	100
LEA	C, S	25

^*∗*^Optimal concentration for binding assays. C = clumps. S = single cells.
